# Interoceptive experiences and ecological care: an embodied approach within therapeutical realms

**DOI:** 10.3389/fpsyg.2024.1246906

**Published:** 2024-02-09

**Authors:** Ximena González-Grandón, Itzel Cadena-Alvear, Melina Gastelum-Vargas

**Affiliations:** ^1^Departamento de Educación, Universidad Iberoamericana, Mexico City, Mexico; ^2^Faculty of Medicine, Universidad Nacional Autónoma de México, Mexico City, Mexico; ^3^Instituto de Filosofía y Ciencias de la Complejidad, Santiago, Chile; ^4^Faculty of Psychology, Universidad Nacional Autónoma de México, Mexico City, Mexico; ^5^Faculty of Philosophy and Literature, Universidad Nacional Autónoma de México, Mexico City, Mexico

**Keywords:** interoception, enactive cognition, therapeutics, ecological psychology, health/illness processes

## Abstract

The conventional dichotomy between human health and disease has historically been approached through reductionist models that emphasize the exclusive causal relevance of physiological and pathological processes. Consequently, self-awareness and affective dimensions, integral to a phenomenological perspective, are often relegated to secondary traits, affording little consideration for the causal role of embodied living organization. Our interest lies in exploring the potential relevance of the phenomenology of embodied self-awareness in relation to interoceptive processes within therapeutic settings. As we illustrate, when the unfolding processes of interoceptive awareness and its affective capacity take precedence, the agent assumes an active, rather than passive, role in their own experience of health or illness. Departing from an enactive, phenomenological, and ecological standpoint, we propose a distinctive perspective on interoceptive processes, relying on an affective conceptualization of a spectrum of experiences of bodily being-in-the-world. Our primary argument posits that considering interoceptive processes from an embodied and ecological viewpoint of the self, interacting with the material and social environment, enables an approach to the gradient of affective experiences of embodied self-awareness—where pleasure or suffering is perceived and felt—in a naturalized, non-reductive, and relational manner. We discern two ways in which interoceptive processes interrelate with the experience of embodied self-awareness: sensitivity (self-affective) and affective-laden perception. Drawing on this distinction, we provide a nuanced description of these experiences within communities of cis-women, exemplified through the contexts of menstruation and endometriosis. This exploration seeks to enhance our understanding of the phenomenology of embodied, ecological, and affective self-experience from within diverse and situated bodies. The goal is to contribute to their autonomy and ability to adapt and self-regulate within therapeutic contexts.

## 1 Introduction

Emerging from a dichotomous value system, daily life events are often framed as either pleasant or unpleasant, comfortable or uncomfortable, healthy or unhealthy, or falling somewhere in between. When an individual experiences illness, the perception of their body is intricately linked to internal processes, shaped by situated interactions and affective valuations (Tsakiris et al., 2011; Maiese, 2014). Notably, scholars like Craig (2009) argue that perceiving one’s body from the internal milieu is a pivotal source of self-awareness, contributing to the regulation of human actions and the maintenance of physical and mental health. We align with this perspective and aim to underscore the importance of internal body perception, emphasizing embodied self-awareness and the role played by interoception, often overlooked in therapeutic settings.

We acknowledge that interoceptive processes constitute an element of the self, extending beyond a mere compartmentalized aspect of the body or the brain. Our reference is to the entire human body, embodying its ecological self as an active agent within the immediate socio-material environment. This encompasses interpersonal relations within ongoing social community exchange and affection. In this regard, we line up with Ulrich Neisser’s proposition that such agents perceive themselves in various aspects, including their location, movement, and actions (Neisser, 1993, p. 4). Also, in alignment with Michel Henry’s self-affectivity (1973), we contemplate a distinct interoceptive sensibility arising from the homeostatic/allostatic aspects of bodily life. Consistent with an ecological and enactive tradition, we refrain from postulating an inner self; rather, we conceive an ecological self as the whole living embodied person as an integral part of a context and community, perceiving, feeling, acting, suffering, and taking responsibility for herself.

Research on the role of interoceptive processes in the whole living experience of an embodied person during affective interactions with the ecological and sociocultural worlds is limited. Habitually, we have embraced localizationist or separatist views that advocate for sharp distinctions between sensorimotor, interoceptive, or affective realms, casting shadows on the unity of the lived body in action and interaction. This perspective has been challenged through phenomenological explorations of experience, where distinctions are found to be blurred. For instance, in the lived experience of interoception, such as hunger, one may feel pain in the intestines, a pervasive negative mood, and an attentional bias toward food signals, compelling movement toward them.

According to the traditional perspective in psychology, which posits our affective life as a result of physiological processes and changes (James, 1890; Damasio, 1994), interoceptive living processes operate as an affective bridge, offering a continuum amid imposed biomedical separations. This viewpoint is further enriched by the insights of enactivism and phenomenology, which consider the self-affective and affective-laden nature of each perceptual experience, leaving the cognitive gap behind. Here, affectivity is construed as the human capacity to regulate and monitor oneself through evaluation in relation to the environment (Colombetti, 2014); while self-affectivity is a more basic self-organized valuation (Henry, 1973). In this context, interoceptive experiences originating from the viscera, the bloodstream, and the living endogenous systems (soft internal organs of the body, such as the lungs, heart, organs of the digestive, excretory, and reproductive systems soaked up by dozens of metabolites and hormones from the blood) also become integral to the ecological, affective and embodied self-awareness, emphasizing the need for a foundational commitment to complete embodiment and organism-environment mutuality.

Moreover, considering the perspectives we adopt, the cultivation of interoceptive embodied self-awareness emerges as a valuable set of skills to acquire within specific contexts. We posit that acquiring the ability to attentively observe our own visceral and endogenous bodily processes can lead to heightened recognition of interoception’s influence on shaping a diverse range of real-time experiences. For instance, this could involve recognizing the correlation between feeling anxious about your child’s first day of school and sensing the subtle rumbling in one’s intestines and creating the habit of taking savile^1^ on an empty stomach at the beginning of each school year. Through such practices, there is potential for enhanced management of self-regulation and a deeper understanding of self-awareness in both its embodied and ecological dimensions.

Indeed, this capacity will enable us to foster interoceptive agency, in short, the ability to actively engage with the possibilities of existence in the world through the cultivation of new habits marked by heightened attention and self-awareness of our own bodies in interaction. This proficiency enables us, on one hand, to interpret categories like ‘health/dysfunction’ in a manner closer to a spectrum or gradient of diverse ways of experiencing embodiment. Where this continuum—referred here to experiences of being-in-the-world—emerges as intricate, dynamic processes that resist reduction to mere function/dysfunction, health/illness or exterior/interior dichotomies. And, it establishes connections with the roles assumed in the human system, where individuals live as agents, patients, and participants involved in their ecological self-regulation.

On the other hand, this newfound ability implies a genuine consideration of the concept of “self-care” and an ethics of care. This involves acknowledging the responsibility of the individual as an agent for the management and control of their individual or shared experiences.

To construct this integrative narrative, the paper is structured as follows: the initial section delves into the functional discourse prevalent in medicine, anchored in health/disease and function/dysfunction dichotomies. This exploration opens the door to considering a spectrum of bodily ways of being-in-the-world. The subsequent section introduces the adopted relational, affective, and perceptual approach to illustrate how interoceptive processes organize in human beings, presenting a self-affective and ecological experience of embodied self-awareness. Various dimensions are elaborated upon in the third section. The fourth section exemplifies the role of these interoceptive experiences in the spectrum of bodily ways of being-in-the-world. Here, we examine the proposed frameworks through the lens of communities of women who experience menstruation and/or grapple with specific painful conditions like endometriosis. In both instances, women can emerge as embodied self-aware experts, navigating these bodily situations, particularly within the context of living through their menstrual cycles. Concluding the paper, we trace guiding actions and revisit certain theoretical accounts to develop more ethical therapeutic approaches. These approaches recognize interoceptive embodied self-awareness as foundational to autonomy, responsibility, and collaborative self-care with others.

## 2 From health/disease to the spectrum of experiences of bodily being-in-the-world

In contemporary discussions within the philosophy of medicine, the definitions of “health” and “disease” are subjects of ongoing debate, with normativist, constructivist, and naturalist approaches being prominent (Khushf, 2007). Normativists posit that these terms entail value judgments, where states of health are deemed desirable and diseased states are to be avoided. In contrast, naturalists seek a scientific and objective definition grounded in biological norms and what is considered natural for human organisms. This approach involves considerations of symptoms and signs to naturalize diseases, characterizing disease as the failure of a part of the organism to perform its normal biological functions.

According to naturalist theories, the concept of ‘biological function’ is seen as normative, emphasizing what should be the case—taking a specific bodily-cognitive reference point as ‘normal’—rather than focusing on what actually is the case. Influential naturalist thinkers, such as Boorse (1977), define “disease” as the statistically abnormal functioning of a specific trait compared to the average functioning of traits within a particular reference class. This naturalist perspective has held sway in the philosophy of medicine in recent decades.

We refrain from relying on bio-statistical criteria to delineate between the normal or typical, disease or dysfunction, symptomatic or asymptomatic. Instead, our focus is on accentuating the diversity inherent in human experiences of being-in-the-world. Our approach, grounded in an enactive and ecological perspective, sought to increase sensibility and skill through self-awareness in interactive dynamics.

In this viewpoint, ‘illness’ is conceptualized as a subjectively felt and interpreted undesirable state, encompassing subjective feeling states such as pain or weakness. It involves perceptions of the inadequacy of one’s own bodily functioning, brought into individual consciousness through dialog with normative and cultural constructs. As a result, this experience is both meaningful and felt (Kleinman, 1992) within the context of interactions with the sociocultural environment.

Embedded within the political agenda of combating social inequalities, communities of philosophers and thinkers on health and disease have turned their attention to understanding the intricate relationship between health, affectivity, society, intersubjectivity, and contextual meaning. The aim is to establish guidelines for action (WHO, 2008). While not delving extensively into these debates here, it is crucial to note that Latin American socio-communitary medicine perspectives have given rise to the idea of reimagining more intricate models to comprehend the phenomena of health and disease where the ecological self is included.

In conceptualizing the health/illness/care process, the objective has shifted toward aligning with the needs of diverse social groups and fostering a different notion of health. This notion transcends the binary opposition of health and disease, making room for a multitude of meaningful ways of being-in-the-world that contribute to an ecological sense of embodied awareness of being. Situated concepts from Latin America, also trying to broaden the concept of well-being, refer to these meaningful ways of being-in-the-world as “good living” (in Spanish: *“buen vivir”*^2^; Acosta, 2010; Zaragocin, 2017) or *“vivir sabroso”*^3^ (Quiceno-Toro, 2016). Therefore, the theoretical-practical change advocated by Latin American Social Medicine and Community Health involves assuming health and life as dynamic and collective processes, without neglecting disease. It includes incorporating explanatory and comprehensive metaphors beyond the positivist paradigm and developing ethical and caring practices that integrate diverse actors and powers (Granda, 2004).

Furthermore, in recent decades, a notable shift has emerged from bioethical and community-oriented perspectives, moving away from a passive view of the relationship between the agent, patient, or client and the healthcare system toward a more independent and self-determining position (Childress, 1990; Maiese, 2021). This reimagining of patient agency can be viewed in two key directions:

Firstly, it encourages individuals to assume greater responsibility for their own lives, empowering them with the right to make autonomous decisions regarding their health experiences. This shift discourages blind obedience to medical advice and advocates for a more collaborative approach to care.

Secondly, it emphasizes the importance of recognizing patients’ subjective experiences, beliefs, and emotional responses to their illness. These factors are seen not only as constraints on certain illness outcomes but also as significant influences on patients’ follow-up and self-management actions (Cai et al., 2023). This holistic understanding of patient agency contributes to a more nuanced and patient-centered approach within the healthcare system.

These rearrangements prompt us to inquire about our ability to recognize and take charge of the feeling of discomfort in our bodies. How equipped are we to cope with ailments? It is paramount for us to accord greater significance to these transitions and advocate for an increased awareness of the phenomenology of ecological and embodied interoceptive self-awareness. Developing this self-awareness can empower individuals to enhance their perceptions of illness, fostering autonomy, self-care, and care for others. Moreover, it can provide valuable insights for healthcare providers, facilitating more specific, empathetic, and accurate assessments. Consequently, this encourages us to genuinely consider and integrate living, adaptive interoceptive experiences within the therapeutic process.

In this context, the conceptualization of the human body and the health-disease process needs to transcend the dualistic-mechanistic vision, which sees living organisms as automatic machines (Merchant, 2006). This view considers organisms as composed of individual parts functioning along linear cause-and-effect chains. In contrast, aligned with our proposal, the enactive and ecological perspective acknowledges the constitutive role of self-organization, and the sociocultural and normative environment in shaping human experiences, particularly considering organizational and dynamical causality in the interoceptive embodied self-awareness. This perspective guides us to recognize the centrality of self-affectivity and affectivity, steering clear of essentialisms and embracing the diversity of relational elements.

It is crucial to acknowledge that as human beings, our predominant interest often lies in orienting our ways of being-in-the-world toward well-being, *“buen vivir,”* or *“vivir sabroso”* rather than predisposing ourselves to suffer from chronic conditions. This nuanced perspective underscores the importance of an integrative approach that considers the interplay of various factors in shaping our experiences of health and well-being.

Building upon these approaches, we can conceptualize the spectrum of bodily being-in-the-world experiences as an interoceptive continuum within the embodied self-awareness of the agent-environment system. This concept originates from a phenomenological perspective that recognizes the interoceptive intervention in the diverse embodied forms of being-in-the-world. It takes into account the myriad feelings, sufferings, and affectivities that unfold within each agent’s life history, intricately woven into the developmental trajectories of each community. In the following sections, we delve into the description of the interoceptive processes that underpin and enable this proposed framework.

## 3 What is interoception from an ecological and enactive perspective?

Surrounded by the contemporary debate on defining interoception (Ceunen et al., 2016), we seek to transcend the conventional perspectives that view it solely as the perceived availability and evaluated relevance of body representations when assessing internal bodily signals (Craig, 2002). We also aim to move beyond the narrow definition that confines interoception to a sensory system detecting input from internal body states through afferent and nerve signals originating from sensors or receptors within visceral organs and the peripheral nervous system (Sherrington, 1906; Craig, 2002). Instead, our exploration delves into the nature of interoceptive phenomenology as an ecological and embodied self-experience starting from the inside, involving “endogenous processes” in connection to its physiological basis, and to their affective, sociocultural, and existential components.

Certainly, broader contemporary definitions encompass interoception as a complex and multi-dimensional process, considering not only the perception of internal bodily signals but also how individuals attend to, evaluate, and reflect on these sensations and their impact on motivation and decision-making (Vaitl, 1996). Current theories in neuroscience further expound on interoceptive perception and evaluation, portraying them as outcomes of a bidirectional interaction between the central, peripheral, and autonomic nervous systems, in conjunction with other physiological systems such as the digestive and cardiovascular systems (Berntson and Khalsa, 2021). These intricate interactions contribute to an integrated bodily sensation, creating a moment-to-moment changing landscape of bodily states for the self (Craig, 2009; Ceunen et al., 2016). Furthermore, this expanded perspective emphasizes the interplay between cognitive, emotional, and physiological elements in shaping the overall experience of interoception.

While neurobiological approaches offer valuable insights, we recognize a potential limitation in their tendency to perpetuate internalism and reductionism (such as Craig, 2009 interoceptive brain maps organized somatotopically). Our proposal diverges by seeking to approach the phenomenological elements of interoceptive processes as a living and interactive phenomenon that can be naturalized without resorting to reductionism. In this way, we conceptualize interoceptive processes as operating from a fundamental affective or value role crucial for life.

We conceive interoception within the lived experience of an ecological self, functioning as an integrated whole within an embodied agent. This occurs when we value, in both a self-affective and affective manner, the ongoing processes within our bodies, intricately intertwined with the surrounding environment. In this section, we aim to elucidate the connection between at least two interrelated dimensions within the domain of interoceptive processes.

The former involves sensibility stemming from the endogenous processes of the organism, independent of conscious perception. In simpler terms, this denotes an interoceptive sensibility emerging from systemic organization, serving as a crucial regulator of self-organization directed toward the persistence and flourishing of the organism. In practical terms, this involves interoceptors sensitive to metabolites circulating throughout our entire endogenous bodies (including the brain, heart, and digestive system through the bloodstream). These interoceptors establish physiological parameters, steering away from perturbations or uncertain interactions, working toward the attainment of a “stable” or “adaptable” state in the homeostasis/allostasis dialog. From an enactive and phenomenological perspective, these fundamental interoceptive elements are integral to biological normativity and basic self-affectivity, shaped by historical and highly specific contexts for particular species and communities.

Interoception is not merely activated as sentience; it also encompasses self-affectivity. This intrinsic self-affectivity, as expounded by Michel Henry—a profound feeling of self or auto-affection that emerges early in the organism’s life and actively influences the physiological parameters requiring maintenance (1973)—demonstrates the establishment of a valuation from an embodied perspective.^4^ Even at these foundational levels, continuous interoceptive sensible assessments unfold during interactions with the environment. This challenges a mechanistic viewpoint that might otherwise perceive the registration and interpretation of internal signals as devoid of feeling.

In the case of humans, this self-affectivity refers to interoceptive self-awareness—an experienced, subjective understanding of being in the world, involving attention, evaluation, and reflection. This encompasses what it feels like to exist in the world from the inside out, gaining particular significance in relation to abilities and contexts (González-Grandón et al., 2021). This lived subjectivity involves an awareness of being sentient or having the ability to recognize one’s interoceptive feelings in a specific way, implying an awareness of the evaluative and affective aspects of these sensations.

Some researchers distinguish this phenomenological level between “interoceptive sensations”—like feeling one’s bladder full or prolonged pain in the upper right abdomen— and “interoceptive feelings”—such as experiencing hunger or thirst. This level is also set apart from the inferential one that compels “interoceptive accuracy”—that is measured through own heartbeat counting—and “interoceptive sensibility”—that is gauged by confidence in one’s interoceptive accuracy (Garfinkel et al., 2015).

For the sake of clarity, we will use the term “interoceptive awareness” or interoceptive embodied self-awareness broadly, referring to being aware of one’s feelings and self’s experiences as the subject rather than the object of those feelings, when interacting and valorating the world. This encompasses both phenomenological and inferential suggested levels. When being-in-the-world we do not confine ourselves solely to interoceptive sensations like thirst, hunger, drowsiness, fatigue, dizziness, or shortness of breath. Instead, we’ll defer the exploration to include the ability to voluntarily shift attention to the endogenous domain interacting with the ecological and social realm, which can involve learning and metacognitive processes, such as feeling the heartbeats, counting them, reflecting on them and relating them to situations in the world. We intend to integrate the interoceptive experiential life with specific valences and motivational roles, aiming to connect so-called interoceptive accuracy with interoceptive sensations or feelings.

We do not deny that certain phenomenological distinctions may be relevant, as between “pre-reflective” or “reflective” interoceptive self-awareness, but they will be very useful in later developments of this research. For the time being this comprehensive approach also enables us to incorporate interoceptive experiences into the learning of interoceptive skills and to recognize them as a sentient starting point of embodied consciousness, echoing William James’ words (1890, 242) about the “feeling of the same old body always there.” After all, we can spontaneously feel our heart beating too fast when climbing a slope or notice our bladder feeling full after drinking large quantities of hibiscus water, and through attentive learning, we can enhance our ability to recognize these sensations, feelings, and inferences more accurately. This involves cultivating the ability to voluntarily focus attention on these interoceptive experiences, day after day, akin to learning new habits, to procure “more precise feeling of the same body always learning and transforming.”

Navigating the delicate task of bridging the gap between valuative and affective subjectivity and its physiological underpinnings constitutes a risky adventure. Nevertheless, the pursuit of uncovering these bridges proves worthwhile, as it sheds light on useful implementations. Indeed, analyzing the interoceptive processes through this integrative lens yields a deeper understanding of its relevance to the field of analysis of embodied experience, particularly in contexts like therapy or accompaniment.

Hereinafter, we will present some ideas that can help to illuminate the intricate interconnections of interoceptive processes between the endogenous realm—encompassing aspects such as hormone circulation, gaseous exchange, or digestive enzyme activity—, with the interoceptive life-world—involving experiences like feeling dizzy on the wheel of fortune or reflecting on the feelings and meanings of orgasm.

### 3.1 Placing bridges between interoceptive dimensions

While avoiding a deep dive into the “cognitive gap” discussion—the problematic thesis that there is an explanatory gap between the phenomenal aspects of the experiences and the material and living processes from which they emerge—, enactive, phenomenological, and ecological approaches offer valuable insights in the exploration linking subjective elements to physiological ones.

The enactive “life-mind continuity thesis,” rooted in systems theory and dynamic theories of complex systems, is grounded in specific concepts, such as adaptivity, autopoiesis and operational closure, and also from a minimal, non-conceptual understanding of mind. It entails a continuity between the self-organizing processes of life with those of the mind. In addition, this thesis also implies that the unit of analysis becomes a process, a system of interdependent processes (Andersen et al., 2000).

In accordance with this thesis, we endorse the processual concept of interoception within the broader and ongoing interaction with the environment. Interoceptive processes operate on a continuum between basic self-affectivity and sociocultural self-concern, evaluating, at each timescale, the minimization or confrontation of uncertainty. When viewing living systems in this manner, each process, including homeostasis/allostasis, bloodstream, attention, awareness, or interactions, unfolds over some timescale or multiple timescales. Understanding how the basic organization on shorter timescales constrains events on longer timescales requires more than analyzing a hierarchy of organizations among units at progressively smaller to higher scales, as in the usual order of community, system, organ, tissues, to cells. Instead, we must consider the strength of interaction with vertical and horizontal layers—a network of connectivity giving rise to emergent phenomena at every level of organization (Bickhard, 2000).

Each interoceptive process involved in human experience operates on diverse timescales. Whether it is the beat of a heart, the persistence of chronic pain, the rhythm of breath, or the development of dietary habits, these processes can span a moment or become lifelong endeavors. This thesis enables us to view the interoceptive process as a unit of analysis, implying a system of interdependent processes in a continuum. These processes occur at different timescales and are dynamically related between the physiological and the attentional, not only in a linear or progressive manner but in constant interaction with the world.

In addition, the enactive concept of autonomy, can also help to bring together these two dimensions of interoception. In this context, autonomy denotes the self-organization and self-maintenance of human beings as systems, mediating their sensorimotor and cognitive dynamics while participating in physical and collective social processes (Barandiaran, 2017). Additionally, the conceptualization of self-organizing systems from ecological psychology lends further support, describing entities capable of actively influencing their interactions with the environment to adapt and alter environmental gradients (Turvey and Carello, 2012). The idea of organism-environment coupling runs parallel to this. Both conceptual frameworks can shed light on how interoceptive sensibility and awareness, as a continuum, are intricately woven into human agency and intentional actions within the broader socio-environmental context.

Phenomenology when granting the capacity to address the conditioning of consciousness, in our bodies and in the world we share with others, is building toward closing the gap. Henry’s self-affectivity builds upon Husserl’s descriptions of the lived-body (*Leib*), to give more weight to the concept of becoming enfleshed with the world: with incarnation coming to the forefront. The lived body, as a locus of primary self-affectivity, is crucial to achieve continuity. Husserl characterizes the lived body as a “null point” (Husserl, 1913), a central organizing principle around which life unfolds. It serves as a foundational basis for bodily awareness, self-affectivity, and affective experiences. Within this framework, interoception operates as a mediating system finely attuned to the environment, contributing to the valuation and adaptation of the lived experience, working from the grassroots to different processes and time scales.

Now, as the concept of the “lived body” acknowledges the necessity of assigning a more substantial causal role to the affective and interoceptive body, it does so in the sense that it becomes imperative to fortify the notion of a self—a complete individual who both undergoes interactive experiences and continually engages in self-affection and self-awareness. This self is conceived as an embodied agent actively participating in the generation of meaning and evaluation, an ecological self.

Drawing from the ecological tradition, which emphasizes organism-environment mutuality, the experience of oneself is seamlessly integrated with the experience of interacting with the material environment and other beings. Indeed, interoceptive processes are intricately connected to the experience of self, of embodied self-awareness in interaction. Gibson (1979) was the first to emphasize that perceiving the self is an inevitable counterpart of perceiving the environment, stating: “To perceive the world is to co-perceive oneself” (1979, pp. 141). Grene (1993) takes a nuanced perspective, suggesting that “To perceive oneself is, except in very peculiar circumstances, to co-perceive the world (...) to be aware of ourselves (...), is to be aware not only of a product of that world but also of aspects of the world that bear on its production” (pp. 112). Neisser (1993) also incorporates face-to-face interaction into his theoretical framework of self-awareness. Grene’s perspective resonates more specifically with our proposal, encapsulating the interconnectedness between perceiving oneself and the world in the context of (ecological) embodied self-awareness:

But even his knowing that, in the examples he cites, are related, at one remove, to the learning of skills, and they are certainly social: how mommy bakes cookies, for example, or how daddy (bless his modern, well-trained heart) goes to buy diapers. These seem to be examples of a small person locating him - or herself in his-her environment, both social-personal and local-ecological. And it is through just such processes of orientation that the historical self is achieved (Grene, 1993, pp. 115).

So, the interoceptive processes, functioning on both the former level by eliminating toxins and waste that eventually exit the body through urine and feces, and on the latter conscious level as embodied self-awareness experiences—such as mastering the control of one’s heartbeat to pass a polygraph test—constitute a continuous series of interactions between living bodies and the dynamic ecological niche. The environmental setting—whether it be light or dark, tropical or winter weather, or the nature of the community, whether friendly or discriminatory—continuously shapes both systems reciprocally on different timescales, forming a “complementary mutually shaping relationship” (Gastelum, 2020; Hernández-Ochoa et al., 2023).

In this context, we argue against a rigid division between the endogenous and exogenous environment, when describing interoceptive living processes.

While various perspectives exist regarding the similarities or differences between the enactive and ecological viewpoints, we, along with fellow researchers, contend that these perspectives can offer complementary ideas that prove fruitful for the objectives of this article. The so-called ecological-enactive perspective (Rietveld et al., 2018) is grounded in three fundamental ideas. Firstly, they emphasize the circular and two-way relationship between perceptual experience and the world as constitutive (Gibson, 1979; Varela et al., 1991). Secondly, they adopt an action-oriented perspective, asserting that perception and action, despite being mediated by distinct processes and pathways, are coupled by ecological laws to regulate behavior adaptively (Varela et al., 1991; Warren, 2006; González-Grandón and Froese, 2018). Thirdly, both perspectives employ dynamical systems theory as a framework to understand experience in the interactions between different levels of organization.

These frameworks collectively enable us to continue narrowing the gap, and to conceptualize interoceptive affect-laden perception as a skill that can be cultivated through interventions or training. This skilled form of embodied self-awareness involves modifying the role of attention to the intricate bodily viscera in situated interaction, for instance, leading to enhanced autonomy and, gradually, to self-care. For example, training to become aware of breathing prior to a presentation can help manage stage fright, or deciding to travel less can be driven by an awareness of the negative impact on intestinal flora and microbiome. As Noë (2004, pp. 2) asserts, perception is a “species of skillful bodily activity.” We suggest delving further into this concept, that interoceptive processes involved in ecological and embodied self-awareness are a form of perception-action coupling, skills that are honed through action.

Importantly, this understanding suggests potential therapeutic applications, recognizing the intrinsic role of interoception in maintaining and enhancing overall health gradients when interacting. For example, some communities of women embody different expectations and habits related to food patterns, coordinating their nutritional practices with the ovulatory cycle to enhance and stabilize their physiological cycles (especially when living with Polycystic Ovary Syndrome; Xenou and Kleanthi, 2021).

### 3.2 Varieties of interoceptive embodied and ecological self-awareness experiences

We propose to distinguish these nuanced interoceptive experiences, characterized by awareness and affective valuation, interwoven physiology with the material and sociocultural environment as embodied expressions of being-in-the-world, in the following manner:

a) *Interoceptive self-affective sensibility* involves basic regulative pre-reflective processes occurring below the level of the agent’s consciousness. It enables the biological organization to establish physiologically viable ranges preferable for self-maintenance, which vary based on the agent’s life trajectories and their environmental coupling history. These processes are associated with visceral activity, the peripheral and autonomic nervous systems, as well as homeostasis/allostasis feedback, contributing to circular and dynamical self-organization. They work to provide viable conditions that cannot be fully generated internally because adaptive changes must be accomplished via interaction with the environment. On this basic self-affective level, a spectrum of stability occurs when the system adapts to an actual perturbation. However, given its dynamic nature, it may enter an unstable state at some point in the future. Indeed, this kind of low-level sensing and self-affectivity processes are necessary processes that contribute to emergent conscious processes, physiological, living and self-organized systems that run in parallel with awareness.
b) *Interoceptive (embodied and ecological) self-awareness:* This level involves the reflective aspect of agents attempting to preserve their own identity. In this interoceptive dimension, individuals engage in evaluative experiences and appraisals of their (self-)bodily awareness while interacting with the world. It encompasses perceptual awareness, emotions, and conscious reflection on bodily self-experiences, as well as the development of skilled possibilities for self-regulation or self-care. Embodied awareness at this level may involve paying attention to breathing, heartbeat, or gradients of pain in the womb, for instance, as forms of embodied affection. It concerns how bodies are present to themselves, how they are felt, and how they relate to the world to undergo transformative experiences.

We will distinguish this conscious experience in an operational rather than ontological way to elucidate how it establishes its relational aspect:

(b.1) The *sociocultural existential-vulnerable relation*, and (b.2) the *ecological and skilled relation*. These relations elucidate how diverse individuals generate meaningful and evaluative interoceptive experiences in a rich material and sociocultural world, grounded in their biological needs and affectivities. Shaped by existential values, sociocultural norms, and opportunities to skillfully cultivate these experiences, individuals transform their ways of being-in-the-world. Embodied self-awareness of experiences like suffering, discomfort, pain, or pleasure is species-specific, biological, and socioculturally normative, experienced subjectively and intersubjectively. Subsequently, both relations describe the potential for transforming one’s embodied self-experience by making interoceptive awareness explicit, fostering alternative forms of agency, and cultivating new habits.

These distinctions, which will be elaborated further below, prompt us to contemplate various modes of being-in-the-world through ecological and embodied self-awareness in material and socially diverse contexts.

a) **Interoceptive self-affective sensibility**

This foundational level involves active processes that continuously adapt and self-organize in response to themselves, the environment and other circumstances.

The naturalization of interoceptive sensibility as a basic self-affectivity can be comprehended through the self-organization inherent in a living body. This involves the continuous monitoring and regulation of the endogenous milieu, achieved through visceral sensations originating from autonomic organs (via descending innervation from the parasympathetic and sympathetic nervous systems through cardiovascular, respiratory, or gastrointestinal systems), humoral secretion from the endocrine and immune systems, and vegetative reactions involving numerous metabolites and hormones in the bloodstream that activate diverse value processes (Fuchs, 2017). This intricate system contributes to the maintenance and constant adaptation facilitated by chemoreceptors, thermoreceptors, and nerve endings from organs. However, this fundamental interoceptive activation is perpetually engaged in a dynamic and fluctuating interaction with external/exogenous processes. In fact, the preservation of physical parameters within the endogenous milieu, such as temperature, pH, and nutrient levels, is always in equilibrium with external factors, including night/day rhythms or the amount of oxygen in the surrounding atmosphere.

This foundational interoceptive sensibility and self-affection plays a crucial role in the ongoing assessment, contributing both adaptive and non-adaptively to restore or not the necessary stability when viability is challenged. In enactive terms, this involves a structural coupling between the organism and its context (Di Paolo et al., 2017).

These processes are influenced by the body’s internal physiological state, particularly in maintaining homeostatic and allostatic balance. The traditional concept of “homeostasis” by Walter Cannon posits an ideal set of conditions for maintaining the stability of an endogenous environment. However, this understanding evolved with the notion of allostasis, acknowledging that there is no single ideal set of steady-state conditions in life (McEwen and Wingfield, 2010).

As conceptualized by McEwen (2000), allostasis is an active process of maintaining homeostasis and a mechanism by which the body restores balance in response to uncertainty. McEwen and Wingfield expanded the concept of allostatic regulation to include adjustments of set points that anticipate cyclic changes across various timeframes. These adaptations may manifest the significant influence of the constitutive relationship with the environment, as seasonal variations in physiology and behavior in preparation for hibernation, migration, or when encountering unexpected threats in a constantly changing and stressful surroundings (McEwen and Wingfield, 2010).

From the perspective we advocate, the viability of the human living system is in constant flux. This stands in contrast to more rigid ideas, such as those that consider healthy processes intrinsically ‘good,’ aligning with physiological processes maintaining life through homeostasis. Conversely, processes deviating from this range are considered intrinsically ‘bad’—for instance, damaged tissue resulting from disease—or, at the very least, not preferable due to their challenging and uncertain nature. Accounting for a basic affectivity where various processes on different timescales self-organize implies overcoming dichotomies, and considering organizations and processes aimed at minimizing uncertainty.

There is ample evidence supporting these non-reductive naturalizations in ecological interaction concerning the “interoceptive self-affective sensibility” in the ongoing process of self-regulation and adaptation. For instance, the high vulnerability to environmental influences on the nervous system, especially during the first years of life, highlights how environmental interactions permanently modify the structure and function of the nervous system through epigenetic changes, particularly through neuroplasticity. Or, in similar discussions, Christoff et al. (2011) refer to homeostatic regulation, in which self-specifying afferent/efferent processes would be central. In their words: “afferent signals conveying information about the organism’s internal state are continually coupled with corresponding efferent regulatory processes that keep afferent parameters within a tight domain of possible values” (2011, 106).

Alternatively, it is also applicable to certain diseases, such as asbestos-related lung cancer. This condition appears to be linked to a prolonged history of exposure to asbestos, whether at work or at home, involving continuous inhalation of its fibers. These fibers lodge in the respiratory system, causing scarring and inflammation, altering breathing patterns and resulting in experiences of suffering. In Leder’s words, when referring to the “exterior interior,” “our body is turned inside out, so to speak. Interoception is shaped by the information we receive from outside sources” (Leder, 2018, pp. 315).

b) **Interoceptive (embodied and ecological) self-awareness**

From a phenomenological approach, this level can be described from a first-person perspective as the regulation of the diverse embodied forms of being-in-the-world. It involves the recognition, evaluation, and regulation of interoceptive experiences. In this sense, we propose that the conscious perception and regulation of suffering, painful, or uncomfortable experiences do not merely stand in a causal correlation to organic dysfunction. Instead, we embrace an organizational and dynamic causality.

Embodied self-awareness experiences may be carrying out their perceptual and regulatory work in a more latent way when displaying their habitual character, remaining invisible in the background of our attentive field—a pre-reflexive self-awareness. However, interoceptive conscious perception, a reflexive self-awareness, becomes more activated and attended to when an obstacle, limitation, or painful experience impedes agents from pursuing a project. Legrand (2007) notes that the healthy body offers a kind of primary being-at-home, which turns into a not-being-at-home with one’s embodiment during states of illness. We can examine how agents perceive the spectrum of bodily being-in-the-world, finding meaning and regulation in their spectrum of suffering and joy within the broader context of their lived experience, which is, in turn, shaped by the possibility of transformation throughout the trajectories of the embodied self and the community (Cadena-Alvear and Gastelum-Vargas, 2022).

When a person feels sick, this experience is not neutral; it is imbued with significance and laden with contextual values. Colombetti (2014) describes the human affective capacity to make sense of the world based on what is salient or relevant, emphasizing the critical role of interoception in shaping our usual sense of embodied self.

In this perspective, the view of interoceptive affective processes is not merely ecological in a material sense but is also sociocultural. Face-to-face or body-to-body interactions establish an ecological and embodied self through interpersonal and inter-bodily dimensions. This existential facet refers interoceptive processes to the patient’s own personal, affect-laden shared perception of their spectrum of pain, which includes intersubjective interpretations, such as beliefs about illness, expected consequences, or whether the spectrum of pain is self-manageable or requires clinical care (Sturmberg and Lanham, 2014).

So, interoceptive awareness in the diverse experiences of being-in- the-world –from suffering to the pleasure of “vivir sabroso” and everything in between–, can become a powerful motivator for agency, intentional actions, and responsible and ethical decisions.

As we said before, we will separate these dimensions to explain them, although ontologically they are entangled.

**b.1)** The sociocultural existential-vulnerable relation, within the enactive framework, focuses on the affective experience of how individuals relate to themselves, their embodied experiences, their lived situation, and their connections with others (De Haan, 2020). In this context, spectrums of pain, as experienced in embodied and ecological self-awareness on an interoceptive and affective level, are influenced by past experiences with similar situations, the imitation of norms from siblings or community members, and the incorporation of ideas about illness learned through various sources such as television, social media, or narratives from parents and physicians. It emphasizes that interoceptive embodied self-awareness experiences are not solely determined by objective medical dichotomies but are deeply entwined with the socio-cultural context and personal narratives.

The phenomenological analysis delves into the intricate relationship between pain and existential concerns such as meaning, body, and identity. Jaspers (1971) concept of “limit situations” sheds light on how experiences of embodied vulnerability contribute to suffering or discomfort when faced with impactful, meaningful, and affect-laden encounters with others. According to Merleau-Ponty (1964), existential vulnerability encompasses the fundamental fragility, openness, and contingency of human existence, emphasizing our interconnectedness with the world and others. Vulnerability, in this view, is not a weakness but a human quality that enables meaningful engagement and transformation in the world.

Luna (2009, pp.133) further expands on the notion of vulnerability, presenting it as a complex and flexible concept rather than a rigid and monolithic experience. Luna introduces the metaphor of “layered vulnerability,” suggesting that vulnerability is a multifaceted and variable phenomenon that can be understood as having different layers, each of which can be addressed or peeled away. This perspective emphasizes the dynamic and nuanced nature of vulnerability, acknowledging its varied dimensions and the potential for transformation through a layered understanding.

Merleau-Ponty, Jasper, and Luna propose that the embodied experience of recognizing oneself as “being a person at the limit” offers an opportunity for self-realization and freedom. This perspective does not negate the mutual influence between our bodies and the surrounding world. By grounding ourselves in this particular experience, we gain the ability to reflect on our circumstances and actively work toward changing them. The acknowledgment of vulnerability and the existential aspects of being-in-the-world becomes a foundation for transformative self-awareness and agency.

In critical and non-critical situations, the ability to be bodily aware, self-regulate, and transform ways of being-in-the-world becomes crucial in reorganizing the world according to the agent’s choice, involving the recognition and transcendence of vulnerabilities.

This reflection opens up a different path toward a new therapeutic horizon by cultivating embodied self-awareness for increased agency, autonomy, and responsibility. In this context, humans bear responsibility for the values and goals that structure their lived experiences. This approach challenges the passivity of the people who suffer certain conditions, when they do not take responsibility for their healthy or unhealthy experience of being-in-the-world, frequently associated with traditional biomedical institutions and leaving little room for their own agency and experience in the proposed treatments.

**b.2)** At this level, we delve into the ecological and skilled relation, which directly shapes perceptual awareness and regulatory processes in interoceptive experiences. Human organisms play an active and vital role in their conscious processes across different time scales. The interoceptive perceptual experience happening in the ecological and embodied self, from the inside, is intricately connected to skillful and effective embodied possibilities for regulation, valoration and action. In the next section, we will explore these ecological and skilled relational experiences in greater detail.

### 3.3 Skilled interoceptive bodily self-experiences and re-learning habits

The conscious level of interoceptive self-awareness, as described earlier, also implies considering experiences as skills. In line with the ecological perspective, humans need to master specific skills in particular environments to have different action possibilities and to be in the world at their best (Di Paolo et al., 2017; Rietveld et al., 2018). Interoceptive conscious experiences can be characterized as learned skills developed through a history of interactions within sociocultural practices in specific contexts. These abilities enable organisms to perceive and voluntarily pay more or less attention to interoceptive or coupling experiences in everyday life but also within therapeutic spaces.

In contemporary times, various approaches to interoceptive training have emerged. These interventions channel attention resources toward interoceptive body sensations, such as the breath, heartbeat, or digestion. Alternatively, attention may be directed toward specific areas of the body, such as the center of the abdomen or the uterus.

An illustrative example of such exercises is provided by Pearson (2019, pp. 37), where he guides an interoceptive practice of body scanning for subtle non-pain to painful sensations.

“Close your eyes and choose to listen to the sound of your breath. Take a few breaths, listening to what your breath sounds like (...) You can do the same with the pain. Decrease how much your brain attends to the painful area by paying less attention to it and more attention to something else, on purpose.”

In the case of women experiencing challenges with sexual desire, an eight-session mindfulness (in-depth attention to internal processes) intervention led to improvements, with the increase in self-reported interoceptive awareness mediating the positive outcomes (Paterson et al., 2017). Similar encouraging results were observed in a nonclinical female sample undergoing a twelve-week mindfulness intervention (Silverstein et al., 2011). Seth et al. (2012) propose that these interventions work because, as the embodied self becomes more fully realized through awareness of ongoing interoceptive interactions, two complementary senses emerge: (i) presence, referring to one’s connection to the moment, and (ii) agency, reflecting one’s ability to effect change. However, these interventions still primarily focus on internal contexts of interoception.

An intriguing question arises: How much could we enhance this type of therapy or support if we earnestly considered the whole person within their constitutive relationship with the world?

Embodied and ecological interoceptive awareness training goes beyond offering an opportunity to differentiate, enhance, or redirect attention to the body. It should encompass an awareness of interactions, such as socio-cultural norms within one’s community, the moments of the circadian cycle, or past coupling experiences. The phenomenological characterization of interoception, as we have proposed, serves as a guide for the potential development of greater skill in embodied self-awareness. However, this goes beyond mere awareness of interoceptive body sensations like hunger, thirst, orgasms, reflections, or habits. Instead, it involves recognizing that experiences within the spectrum of pain and joyful states occur in specific situations and contexts, influencing our bodily awareness.

Thus, training interoceptive ability involves cultivating endogenous bodily awareness and contemplative attention to become more aware—or less aware—of interoceptive processes interacting in particular environments. Being aware of the escalation of my urge to pee in the presence of a clean bathroom nearby, or recognizing the twinge in my abdomen when confronted with an injustice before my eyes. In uncomfortable circumstances, increased awareness may lead to more suffering in some individuals, while in others, it might have the opposite effect. Discernment from the perspective of the person as an ecological self is crucial in such practices, which require attention to the body in interaction.

For instance, interoceptive ecological and embodied self-awareness of vulnerability can serve as a practice for cultivating forms of agency, especially when a person confronts distressing or uncomfortable feelings that may manifest as painful experiences. Individuals may seek relief by recognizing vulnerability, for instance, skillfully employing ecological and interoceptive agency to regulate or eliminate the discomfort arising from within or outside their embodied selves, and also assigning value and meaning to these experiences based on historical and sociocultural factors.

Indeed, this interoceptive practice ensures a conscious transparency of embodied feelings and action possibilities, facilitating expected conclusions or restorative interactions within a world of bodies and shared meanings (González-Grandón, 2023).

Understanding skilled interoceptive experiences in this way, provides insights into everyday instances of adaptation, such as the re-learning of normal regulatory patterns post-injury, body reconfiguration, or adjusting to radical changes in our environment. In therapeutic contexts, there is a potential to guide individuals in assimilating actions to new situations based on heightened interoceptive embodied and ecological self-awareness.

The emphasis of these adaptive networks should revolve around the restoration of continuous self-regulation and self-care in connection with the material and social realm, ultimately contributing to flourishing. Participating in new regulatory patterns, such as listening to and attempting to synchronize with your partner’s heart and breathing patterns during joint actions, not only holds potential biological implications on homeostatic and allostasis cycle setpoints but also carries potential socioaffective implications for both self-care and the care of others. This dynamic aspect of the interoceptive process is accentuated when these interactions become habitual.

On the flip side, specific relationships with the environment can be pivotal in enabling agency, acting as a causal element in regulation by providing relief from physical symptoms or trauma. May (1958) elucidates how the connection with natural landscapes can cultivate awareness experiences of well-being, a notion echoed in disciplines such as Medical Geography, Environmental Psychology, or Horticultural Therapy (Jiang, 2014). Taking it a step further, Krueger (2021) argues that atmospheres actively shape experience and regulate affectivity and behavior. Beyond offering affective color, atmospheres provide affective and action possibilities, influencing the ways we connect with others. This perspective highlights the therapeutic potential of delving into the role of environments and atmospheres in harmonizing with interoceptive embodied and ecological self-awareness.

Further, examining the social realm reveals how social norms can alter embodied interoceptive experiences. The structures within the social environment are perpetuated in individuals’ habitus (Thorpe, 2009). For example, women historically have faced impediments to full agency in various societies, and communities have developed taboos and stigmas ingrained in the habitus of different societies, limiting women and other groups from various aspects of socio-cultural life. As Thorpe (2009) notes, “the habitus-field-complex”^5^ illustrates the synchronous nature of constraints and freedom that women live during their life cycle. Within each habitus-field complex, agents either transform or uphold the configurations of relations.

The descriptions we have formulated of interoceptive self-awareness experiences and their analysis of perceptual and self-regulation in the spectrum of bodily being-in-the-world work as a broad approach that can be applied to human beings in general. However, below we want to illustrate interoceptive self-awareness experiences by applying them to communities of women.

## 4 Situating women’s being-in-the-world experiences: menstruation and endometriosis

The case of women is particularly significant, considering their co-constitution as embodied agents influenced by sociocultural norms that shape their gradients of pain and pleasure as beings-in-the-world throughout their embodied physiological cyclicity. In the following sections, we will illustrate how our framework of interoceptive embodied self-awareness can be applied to the case of communities of women.^6^

Let us embark on an exploration of a fundamental aspect we have identified—the timescales of basic interoceptive self-affectivity within communities of women. In these communities, women act as living agents navigating uncertainty, engaging in self-organization, and adapting to their surroundings. The basic physiology of the menstrual cycle is a complex and coordinated sequence of events involving hormones and other metabolites circulating in the bloodstream from glands such as the hypothalamus or anterior pituitary toward, at least, the ovary and endometrium. The length of the menstrual cycle varies among these communities, ranging between 21 and 40 days, with a pancultural average of 28 days.

In medical sciences, the ovulation phenomenon divides the menstrual cycle into two distinct phases. During the first half (known as the follicular or estrogenic phase), an egg develops due to an increase in the secretion of hormones into the bloodstream, especially hormone estradiol in large quantities. In the second half (luteal or progestational phase), various living processes help to prepare the womb for the implantation of a developing embryo through the development of the endometrium. From our perspective, the living systems involved in this process extend beyond the mere influence of hormones on a target organ; they resemble an orchestra of cardiac, digestive, respiratory, and proprioceptive activations. Indeed, these systems are in a constant state of perturbation due to environmental factors across various timescales—be it travel, stress, exercise, or eating habits. In this dynamic context, basic interoceptive processes persistently evaluate interactions and drive specific changes within this basic dimension.

Turning to the dimension of interoceptive experience as playing a pivotal role in shaping their perception of the world, we take into account the sociocultural and material elements that surround women’s life. Layers of vulnerability, including class, racialization, and ethnicity, intersect with these experiences. Consequently, institutions such as the modern biomedical system and Western cultural narratives act as regulators of behavior and facilitators of interoceptive embodied self-awareness, affecting emotions and action possibilities. These factors shape, impact, and differentiate women’s situations in diverse regions (De Jaegher, 2013). Some narratives and sociocultural norms constrain women’s interoceptive embodied and ecological self-awareness and their approach to relational self-care or medical care, such as the devaluation of their suffering and experiences of spectrums of pain, such as persistent pelvic pain or menstrual-related disorders. Norma Blazquez highlights how, in the case of hypersexuality (formerly called nymphomania), “scientific knowledge, particularly biomedicine, has used devices to exercise control over women’s sexuality and reproduction, affecting their sensitivity and capability to know themselves” (Blázquez, 2011, pp. 82). The impact of these narratives has led to invasive medical procedures such as forced sterilization, clitoridectomy, and hysterectomy, as well as limited research on bodily processes in women.

Cultural narratives and sociocultural norms can, however, have a beneficial impact on interoceptive experiences by providing the normative and evaluative basis for incorporating new meanings, habits, and skills. This includes forms of embodied self-awareness, as well as the acquisition of embodied practices that are attentive and shared.

In specific contexts entrenched in a gendered value system that reinforces the healthy-unhealthy dichotomy, certain bodies are deemed intrinsically abnormal or deviated, consequently being considered less worthy of a dignified life. In this regard, experiences of being-in-the-world^7^ that impact the bodies of cis-women and feminine-non-conforming persons have been framed as dysfunctional or abnormal. This perspective often departs from a reductionist view that does not adequately consider the phenomenological components of these experiences.

Accordingly, the intersubjective encounter between cis-women and gender-non-conforming individuals^8^ in the healthcare environment can wield a significant influence to re-elaborate self-caring skilled interoceptive experiences. While we initially focus on cis-women to discuss interoceptive embodied self-awareness within the spectrum of possible states of pain, we recognize the importance of extending this approach to other diverse communities that have been systematically marginalized.

To offer a more vivid understanding of interoceptive-ecological being-in-the-world experiences in the lives of cis-women, we briefly illustrate the internalized social constraints that influence their lived experiences of suffering in specific bodily situations, such as menstruation and endometriosis.

### 4.1 Menstruation

Menstruation is a bodily phenomenon which can be described from various time scales, diverse processes between physiology and meaningful experience that are entangled with implications from our environment and society, where spaces, expectations, and daily activities do not always accommodate cyclic menstrual bodies. Iris M. Young describes a “somatophobic culture,” where bodily processes like menstruation are deemed dirty and frightening. In such instances, women seeking medical advice for menstrual pain symptoms are often dismissed with statements like “it’s part of being a woman” (Young, 2005). This prevailing narrative intricately woven into specific intersubjective and sociocultural discourses, where various artifacts and normative spaces can subject and discipline feminized bodies to shame, abjection and the medicalization of cis-women’s pain and suffering (Roberts, 2020, pp.177). This invisible stigma permeates the body and leaves tangible imprints on interoceptive embodied self-awareness: “It should not kind of show. It should be, girls should have it, but it should not appear as if they do” (Cecilia, 21 years; Brantelid et al., 2014).

Critical menstrual studies (Young, 2005; Bobel et al., 2020; Helmick, 2020) provide diverse perspectives to understand and challenge the biological phenomenon of menstruation. Johnston-Robledo and Chrisler (2020) emphasize that menstruation, menarche, and menopause are fundamental self-organized valuative processes. These processes involve, among other components, hormones circulating through the bloodstream to different parts of the brain or the female genital system, generating certain regularities in female cycles. However, they can be influenced by a self-disciplining body project, where menstrual hygiene industries and narratives alter their regularity or the way they are experienced interoceptively. In essence, society’s belief that menstrual blood is aversive or an unclean substance affects all dimensions of the experience of being in the menstruating world. This perspective finds further support in first hand testimonies from women of various ages.

“Well, I do not feel as fresh. I think it’s great when your period is finished, finally. That first shower after your period is finished, that feels just great” (Frida, 27 years; Brantelid et al., 2014).

The testimony and the preceding statement exemplify how the conscious experience of embodied self-awareness during menstruation, and also self-affective basic interoceptive processes, are shaped by layered vulnerability rooted in the sociocultural relations established throughout the life trajectory of cis-women (b.1). These relations label menstruation as a ‘dirty’ process or an inherently uncomfortable experience. Moreover, the ecological and skilled relation comes into play as women employ various self-care strategies to navigate the potential obstacles and spectrum of suffering within their sociocultural contexts where menstruation is not universally accepted as a common and natural cyclic process (b.2). This relation (b.2) can also be illustrated by the cases of impoverished menstruating communities that find obstacles to live a dignified menstrual process (e.g., access to menstrual education, toilets, menstrual products, painkillers, etc.).

Socio-cultural artifacts surrounding the premenstrual phase often perpetuate negative stereotypes about cis-women, portraying them as out-of-control, violent, irrational, or physically and mentally ill (Roberts, 2020; Ussher and Perz, 2020). These narrative artifacts play a role in constructing physical restrictions for feminized bodies, disempowering women and girls, limiting their agency and skilled interoceptive experience in the world and over their bodies (Piran, 2020).

Yet, the embodied and ecological self-awareness of skillful interoceptive experience can provide agency creating opportunities that empower cis-women to exercise autonomy, and self-care in managing their menstruation. For instance, in the context of interoceptive menstrual experiences, women may feel the need to catch their breath while preparing for the onset of menstruation. This preparation might involve practices such as getting extra rest, applying heat to areas with local pain, adopting a specific diet to mitigate inflammation associated with heightened menstrual pains, and more. Here, the principle of “exterior/interior” acts as a bridge, connecting interoception between the affected inner body and the motivation behind actions in the external world.

Initiating a counter-culture regarding menstruation may involve acknowledging and promoting menstruation as a vital bodily process and a crucial indicator of overall health and awareness. Advocates of “menstrual activism,” “menstrual justice,” and “menstrual pedagogies” have applied these principles with the aim of fostering social change (Guillo, 2014; García-Huidobro-Munita and Montenegro-González, 2020):

“I wanted to get to know my own body, sort of, and how it really works. Not how the hormones affect it… to feel that you get your period. Somehow, it’s a way of knowing that you sort of have a working menstruation cycle” (Jenny, 25 years in Brantelid et al., 2014).

As mentioned above, changes along the cycle are not only influenced by hormone levels, also, communities of menstrual people experience changes in their cycles when facing disturbed circadian rhythms, continuous stress or sleep perturbations (Baker and Driver, 2007), connecting to the ecological-embodied relations (b) in the self-experience of menstruating communities. These modifications illustrate the intricate dialog between diverse systems and are also manifested in different timescales. These reinterpretations can contribute to the development of diverse therapeutic approaches, knowledge systems, and an ecological-skilled relationship with conscious embodied menstruation as a form of interoceptive self-awareness (b.2).

Menstrual communities could generate strategies to understand and gain awareness around their cyclicity, thus, also coping with modifications like the ones listed above. In section 5 we will connect the previous examples with our proposal toward interoceptive and ecological care.

### 4.2 Endometriosis

Frequently referred to as the “missed disease” or “a riddle wrapped in a mystery inside an enigma” (Guidone, 2020), endometriosis involves the growth of endometrial-like tissue outside the uterus, leading to intense pain and the formation of adhesions and cysts. In this type of conditions the relationship between physiological and experiential dimensions of interoceptive self-affectivity and self-concern is evident. Unfortunately, healthcare professionals and individuals dealing with this condition often trivialize, misdiagnose, or normalize endometriosis (Grundström et al., 2018). Elaine Denny’s work (2003) reveals that some cis-women had their pain experiences dismissed as a common aspect of menstruation rather than being attributed to a specific medical condition: “I had specifically been told that it was just part of being a woman, it’s just one of those things.”

Consequently, those affected might postpone seeking a diagnosis, convinced that their symptoms align with ‘normal menstruation’ or fearing the potential dismissal of their pain as ‘imaginary’ (Bloski and Pierson in Guidone, 2020). In terms of interoceptive embodied awareness, cis-women diagnosed with endometriosis make distinctions between various types of pain based on their experiences. They characterize “normal” pain as manageable with analgesics, while the pain specific to endometriosis elicits more intense descriptions of the spectrum of pain:

“Sometimes it was so bad that I could not move. I was paralyzed with pain… I would say it was like someone knifing you. It felt like there was a knife going into each ovary.” (Denny, 2004, pp. 644; emphasis added).

“The best way I can describe it is if you, sort of, imagine someone with nails clawing inside your stomach. It’s that intense, and it almost comes in waves” (Denny, 2004, pp. 644).

In this first-person testimony, a comprehensive interoceptive phenomenology is vividly expressed, intricately entwined with norms and narratives around pain perception. Additionally, treatments for endometriosis are often invasive and may have unpleasant side effects, offering only short-term relief of symptoms. Unfortunately, affective subjectivity is typically not accorded significant consideration or importance in treatment, potentially leading to gyneco-obstetric violence or less empathetic therapeutic practices.

Cis-women with endometriosis grapple with symptoms that impact their daily functioning across physical, cognitive, and affective domains. They acknowledge the reduction of concentration and attention resources, influenced by societal norms that emphasize productivity within a neoliberal system. The layered vulnerabilities and the habitual-ecological level become even more intricate when considering the diverse contexts in which cis-women live. For instance, in some places in Latin America, these challenges are compounded by heightened social stigma intertwined with complex racial, cultural, and class relations (see Matías-González et al., 2021). These difficulties ground their experiences within a complex sociocultural existential-vulnerable relation (b.1) that influences how they live their condition, for instance, with higher stress, hopelessness to find non-invasive pain managements, or an empathetic care system.

## 5 Interoceptive-ecological therapeutics in autonomy and communitary ethics of care

The framework we have outlined offers insights into the benefits of conceiving interoception as a perceptual and affective ability, paving the way for the development of methodologies that delve deeper into these aspects. The provided examples serve as a roadmap for recognizing the significance of nurturing interoceptive experiences, especially for marginalized human groups. Here, we propose to complement existing methodologies to cultivate interoceptive and ecological embodied self-awareness. This enhancement aims to enrich the diversity of interoceptive experiences within material and sociocultural interactions, thereby amplifying the potential for adaptive self-regulatory actions (Nielsen and Kaszniak, 2006), like in the examples of menstruation and endometriosis revised previously.

To be fair, it is important to consider other therapeutic practices that aim to foster the relational aspect of interoceptive processes. These practices focus on the self-regulation of physiological processes and are often referred to as affective interoceptive practices. Examples of such practices can be found in body–mind approaches like Feldenkrais, Alexander’s method, Somatic Experiencing, Breath Therapy, or Mindful Awareness in Body-oriented Therapy (Kabat-Zinn, 1982).

For instance, Mehling et al. (2012) have suggested a conceptual framework that differentiates four dimensions within the development of attentional interoception. We enhance and expand upon these dimensions, incorporating aspects that consider the ecological self. This comprehensive approach aims to specifically cultivate the interoceptive embodied and ecological self-awareness that we have proposed:

a) Awareness of one’s own body sensations, worries, feelings, vulnerabilities, and movement possibilities, refers to the capacity to increase sensitivity when opening the senses, to discern and identify bodily cues from within, considering the coupling history of interactive experiences.
b) Being aware and actively listening to the situation, involves utilizing all sensory possibilities. This includes awareness of one’s own body sensations in interaction with the current situation, whether it be contemplative, communicative, or a learning environment. Additionally, it entails attunement to the material context, considering factors such as the temperature of the environment, lighting, textures of the floor, environmental shapes, smells, and sounds.
c) Interpersonal awareness involves becoming aware of other human agents around us. It entails avoiding conceptual judgment and, instead, actively listening and negotiating through a non-verbal bodily encounter to attend to the sensations and affectivities of otherness.
d) Skilled attention encompasses the intensity of attention directed toward bodily sensations in interaction with situational and environmental cues, coupled with the capacity to control attention.
e) Attitude of learning interoceptive awareness involves the person’s inclination to trust or worry about bodily cues in interaction, coupled with the learning necessary to be able to rely more fully on them.
f) Mind–body-environment integration encompasses the awareness of how the embodied self-changes with emotional states and involves the perception of a global embodied self in ecological and social interactions. It can include the ability for becoming or transforming when being in the world interacting with the material world and when participating with others.
g) Inter-affectivity and inter-corporeal integration involve learning to pay attention and be aware of the rhythms and patterns of the environment and other people. This includes seeking diverse forms of coordination with elements such as light and dark cycles, affections, breathing frequencies, looks, gestures, and movement patterns.

In this methodology interoceptive embodied and ecological self-awareness, is viewed as a skill to be mastered, enabling individuals to engage with a broader range of possibilities for agency, autonomy, and can be designed to empower individuals in this regard. This perspective acknowledges that existential interoceptive affective subjectivity can be trained through specific abilities within particular contexts, moderated by attentional, volitional, and ecological cues (Bornemann et al., 2015). It emphasizes that interoceptive perception involves not only cultivating attentiveness directed toward oneself but also engaging in inter-bodily interactions with others. For example, interventions involving brief mindfulness training and shared interpersonal narratives have demonstrated efficacy in managing endometriosis-related pain and restoring psychological stability (Moreira et al., 2022). Also, treatments like mindfulness-psychological intervention with practices involving conscious pelvic movement, breathing techniques and empathetic attention to painful areas have shown to reduce pain (Samami et al., 2023).

Embracing skilled interoceptive and ecological awareness when integrating interoceptive-ecological care provides an avenue to discuss caregiving, emphasizing that integrative and holistic healthcare practices should encompass embodied self-awareness strategies and networks of empathetic support. Holistic care practices are aligned with a multidimensional perspective on therapeutics, acknowledging that individuals dealing with painful conditions may benefit from a range of therapeutic and care approaches (De Haan, 2020).

The enactive-ecological approach to therapeutics underscores the crucial role interoceptive experience plays in care actions during painful conditions of being-in-the-world. This approach aligns with a comprehensive understanding of healthcare as a complex system. In this system, care providers, patients, policymakers, local caring environments, and supporting networks collaborate to develop practices that empower patients to become more autonomous and responsible for their embodied actions and perceptions (Sturmberg and Lanham, 2014).

It is imperative to reconsider and collaboratively design therapeutic approaches that incorporate contextual support and training in embodied self-awareness, skilled interoceptive practices, and agency. The concept of an “ethics of care” (Mies and Shiva, 1993; Gilligan, 1995; Young, 2005; Navarro and Gutiérrez, 2018) aligns with the growing acknowledgment of the sustainability of life and caregiving as fundamental human rights. This perspective advocates for shared responsibility and frames caregiving as a public concern (Ángeles and Tena Guerrero, 2014). The ethics of care directs our attention to the involvement and responsiveness of human relationships (Gilligan, 1995), emphasizing the interconnected agency of individuals in sustaining life through mutual care. This approach would also entail adapting therapeutic environments and caregiver practices to be attuned to the intricate “intra-, interpersonal, cultural, historic, and ecological circumstances” of those seeking help (Röhricht et al., 2014).

Other therapeutic avenues to foster interoceptive self-awareness through shared vulnerability for pain management include systemic therapy (Bertrando, 2018), feminist narrative therapy with intersectional methodologies (Guzmán and Martínez, 2014), body psychotherapy (Röhricht et al., 2014), and affective therapeutic techniques that situate affectivity within sociocultural and ecological contexts (Ahmed, 2014; Garzón Ospina and López Sánchez, 2023). These methodologies aim to dissolve the boundaries between intersubjectivity and self-experiences, moving beyond individualized approaches, providing agency, and scrutinizing the sociocultural interconnections that shape experiences of suffering and pleasure. This includes examining conditions of oppression and inequalities that may intensify suffering. It is noted that people who live in unequal conditions (geographic, ethnic, racial, socioeconomic barriers) could experience intensified suffering given their lack of access to healthcare insurance, compassionate gynecological care, diagnosis, or treatments to cope with endometriosis (Fourquet et al., 2019). This is also exemplified in the limited research found about endometriosis lived by racialized cis-women and gender non-conforming bodies that also cope with this condition.

Recognizing and addressing the intersection of different layers of vulnerability is crucial for developing a more ethical approach to addressing the spectrum of pain and suffering throughout human life trajectories via interoceptive embodied and ecological self-awareness experiences.

## 6 Final thoughts

In the framework proposed here, interoceptive processes, encompassing both basic and experiential dimensions, offer the potential to shift from a dichotomy of health/illness toward a non-reductive, naturalized perspective of interoceptive embodied and ecological self-awareness. This shift aims to foster greater agency, autonomy, and an ethics of care within therapeutic environments.

The enactive-ecological perspective allows us to conceptualize interoception as a perceptual and affective skill that can be nurtured within specific contexts. It involves recognizing an experience with its diverse possibilities and cultivating it through self-regulation to establish connections with ourselves, our lived situations, and others. Research on the constitutive role of contextual interactions helps us comprehend the ecological nature of interoception in living human beings.

In a comprehensive sense, multiple layers (physiological, self-affective, experiential, existential, intersubjective, sociocultural) dynamically collaborate in different time scales, giving rise to diverse interoceptive experiences within specific environments. This proposition contributes to addressing the cognitive gap in discussions about the relationship between physiology and subjectivity. Considering embodied interoceptive self-awareness as a complex set of abilities, working broadly across various timescales, including basic self-affective processes, can be instilled through shared processes of re-learning. This approach fosters situated agency through affective learning.

We adopted theoretical frameworks from enactivism, ecological psychology, and phenomenology to articulate an interoceptive and ecological comprehension of the spectrum of bodily being-in-the-world experiences of human agents. This perspective, considering individuals as embodied and ecological selves, transcends dichotomies. Recognizing alternative ways of living, such as “*vivir sabroso,*” we emphasized that experiences of suffering, illness, or health are intricately linked to individuals and contexts. Moreover, these experiences emerge from ongoing interactions across diverse dimensions of interoceptive experience, forming a continuum of varied states rather than discrete entities.

Furthermore, this spectrum of experiences contributes to a more comprehensive understanding of “dysfunctions” or “disorders,” paving the way for the development of integrative therapeutics and an ethics of care. This departure from a (inter-)subjective and interdependent approach contrasts with many clinical traditions in biomedicine that often disconnect the patient’s lived experience from their developmental trajectories. Informed by interoceptive experiences, integrative therapeutics and ethics of care envision alternative lived experiences of endometriosis and menstruation. In these scenarios, caring and sharing experiences empower human beings in general, cis-women and other marginalized embodied and ecological selves to become agents who belong, eliminating the sense of being outsiders due to a pathologized condition in the body, and fostering.

## Author contributions

XG-G, IC-A, and MG-V have written, edited, and discussed together the whole article. All authors contributed to the article and approved the submitted version.

## Footnotes

^1^Savile or aloe vera, taken orally in beverages, controls and relieves symptoms of chronic gastrointestinal problems such as constipation, irritable bowel, gastritis and reflux, as well as functioning as an antiviral and antibacterial agent.

^2^“buen vivir” o “sumak kawsay” has been introduced in the Ecuadorian Constitution since 2008, as a new model of living well. In comparison to the Western, dominant conception, the notion of “buen vivir” implies community coexistence for the originary communities and other human groups, as well as a harmonious relationship between humanity and nature (i.e., “Mother Earth”) through the conservation and ethics of care of the ecosystem (Gudynas, 2011).

^3^“Vivir sabroso,” similar to “living tastefully,” is a conceptual framework of oral thinking communities proposed from the daily heritage of the Colombian pacific, and systematized by the anthropologist Quiceno-Toro (2016). It comes from a long-standing embodied praxis not inscribed in Western canons, about an integral way of conceiving life, a philosophical principle of Afro-Colombian communities, linked to Ubuntu and Muntu. It refers to life in community, to “I am because we are “, to the coexistence of all beings on earth, to generate and to share life, and the aware aspiration to live every experience with taste. Crucially, it is related to embodied pleasant experiences, opening up the possibility of satisfying the desire for a dignified life.

^4^This self-affectivity as elucidated in Henry’s terms, is considered a vital component of interoceptive sensibility. In this perspective, the subject-object dichotomy becomes unnecessary, underscoring the importance of endowing homeostatic/allostatic processes with self-awareness. Henry’s phenomenology of incarnation precisely illustrates how this self-affectivity resists reduction to intentional meaning, maintaining its independence from higher conceptual understanding.

^5^The habitus-field-complex refers to a structured system-in terms of power relations- of social positions occupied by institutions or individuals engaged in the same social activity. “In order for the field to function, there have to be stakes and people prepared to play the game, endowed with the habitus that implies knowledge and recognition of the immanent laws of the field, the stakes, and so on” (Bourdieu and Loïc, 1992, p. 72).

^6^Cis-women are individuals who identify with the feminine gender assigned at birth based on sexual-biological traits. Gender is a multidimensional category to structure social life in terms of roles, identity, expression, desire and subjectivity (Ciccia, 2023). Gender is associated with sexual-biological characteristics such as the external genitalia, endocrinological features, reproductive processes, etc. Feminine-non-conforming persons do not identify with the feminine gender assigned at birth. It is important to name these experiences to make visible the diversity of communities as well as to broaden our comprehension of the current debate on how biological processes associated to sex are in dialog with gendered normativities.

^7^The concept of *being-in-the-world* in these cases is reflected as the state of situated relatedness of women beings to their social and physical world, thus, embodiment is located and in continuous interaction where: “the body is layered with social and historical meaning and is not some primitive matter” (Young, 2005, p. 7).

^8^It is worth noting that menstruation and endometriosis can also be situated in gender-non-conforming individuals (e.g., trans-men, non-binary people), involving higher invisibility due to the lack of awareness and research about their experiences and the assumption of these phenomena as being ‘exclusive’ of cis-gender women.
